# Comparative evaluation of Chlorhexidine, Metronidazole and combination gels on gingivitis: A randomized clinical trial

**DOI:** 10.1016/j.isjp.2019.04.001

**Published:** 2019-04-03

**Authors:** Sheikh Bilal Badar, Kamil Zafar, Robia Ghafoor, Farhan Raza Khan

**Affiliations:** Aga Khan University Hospital, Karachi, Pakistan

**Keywords:** Gingivitis, Dental plaque, Chlorhexidine, Metronidazole

## Abstract

**Introduction:**

Dental plaque has been established as an etiological factor in the development of gingivitis and periodontitis. Several antimicrobials including Chlorhexidine and Metronidazole have been used as an adjuvant to mechanical plaque removal.

**Objective:**

To compare 0.8% Metronidazole gel, 0.2% Chlorhexidine gel and alternate application of the two gels in reducing gingival inflammation when used for 14 days as an adjuvant with home based oral hygiene instructions.

**Methods:**

The bleeding sites, probing depth and the gingival index score will be calculated. (Group A standard 0.2% Chlorhexidine gel applied on the marginal gingiva; Group B subjects will be instructed to apply 0.8% Metronidazole gel; Group C subjects 0.2% Chlorhexidine gel on the marginal gingiva after morning tooth brushing and 0.8% Metronidazole gel after evening tooth brushing). After 2 weeks second clinical examination will be carried out for bleeding sites, probing depth and the gingival index score. Scaling & polishing of teeth in all three groups and oral hygiene instructions will be reinforced. Subjects will be recalled at 6 weeks for evaluation of gingival and oral hygiene indices. Descriptive statistics for variables such as age, baseline clinical parameters including probing depth, bleeding sites, gingival index, and oral hygiene index will be computed. Generalized estimation equation (GEE) will be run to account for the correlated data for the comparison of study arms. Friedman test will be used to assess the bleeding sites among three study groups at the baseline and endpoint. Similarly, gingival index and oral hygiene index in the three groups at baseline, after 2 weeks and 4 weeks will be compared. The level of significance will be kept at 0.05.

## Highlights

• Gingivitis is one of the most prevalent diseases that affects 82% of adult population.
• This current protocol is effective at reducing the gingival inflammation.
• The use of alternate gel application of Chlorhexidine and Metronidazole showed improvement in the treatment of gingivitis.
• Need further implementation of the protocol followed by re-assessment.

## 1 Introduction

Gingivitis is one of the most prevalent diseases that affects 82% of adult population in the United States [1]. Dental plaque has been established as an etiological factor in the development of gingivitis and periodontitis [2]. Plaque is primarily composed of bacteria in a matrix of salivary glycoprotein and extracellular matrix and removal of this bacterial biofilm is the cornerstone in the treatment of gingivitis and periodontitis [3]. Bacterial biofilm is primarily disrupted mechanically by scaling and root planning, however, several studies have shown that mechanical debridement alone sometimes remains insufficient in the removal of flora responsible for the periodontal diseases [4], [5]. Due to this reason chemical plaque removal agents as an adjuvant to mechanical therapy have gained popularity [6], [7], [8].

The ultimate goal is to remove the offending pathogens; therefore, several approaches have been employed to treat gingivitis based on the systemic and topical administration of antimicrobial agents [9], [10]. Since, the prolonged use of systemic antimicrobials can lead to increased antibiotic resistance, nausea and diarrhea; therefore, investigators are now focusing on the development of localized drug delivery systems that can allow maximum concentration on the target site, thus minimizing the potential systemic effects [11], [12].

Several antimicrobials including Chlorhexidine and Metronidazole have been used as an adjuvant to mechanical plaque removal. Chlorhexidine is the gold standard mouthrinse to treat gingivitis. Several studies have proved that adjuvant use of Chlorhexidine does not provide additional benefits on periodontitis. It has s broad antiseptic spectrum and substantivity [13]. It acts by directly altering the integrity of bacterial cell membrane [10], [14], [15]. However, its use is discouraged because of its unpleasant taste and undesirable tooth staining [13].

Metronidazole has been used by several researchers due to its selective antimicrobial activity against the obligate anaerobes. It has been used successfully for the treatment of gingivitis and periodontitis [3]. Meta-analysis has shown the effectiveness of localized use of Metronidazole as an adjunct to scaling and root planning [16]. Miani et al. concluded that the use of Metronidazole gel significantly reduces the total bacterial count in the gingival crevicular fluid [17]. A study conducted by Pradeep et al. [18] showed that both Metronidazole gel and Chlorhexidine gel are effective in the treatment of gingivitis. However, Leiknes et al. indicated that the use of Metronidazole gel does not improve the treatment outcome when used in combination with scaling and root planning [19]. Several chemotherapeutic agents have been compared in the quest for the most effective treatment modality in the cure of various categories of periodontitis. However, there is scarcity of data regarding the effectiveness of Metronidazole and Chlorhexidine gel in the treatment of gingivitis, therefore, it warrants a study.

Since, measurement of the true endpoint of gingivitis and periodontitis is not always possible, therefore, anatomical measurements are considered as the most effective surrogate endpoints for quantifying the periodontal damage [20]. This includes the use of different gingival and periodontal indices to evaluate periodontal diseases [21], [22], [23].

### 1.1 Null hypothesis

0.8% Metronidazole gel, 0.2% Chlorhexidine gel and combination of both Metronidazole and Chlorhexidine gels are equally effective in reducing gingival inflammation.

## 2 Methods

### 2.1 Objective

To compare 0.8% Metronidazole gel, 0.2% Chlorhexidine gel and alternate application of the two gels in reducing gingival inflammation when used for 14 days as an adjuvant with home based oral hygiene instructions.

#### 2.1.1 Primary outcome measure

Bleeding index (BOP in percentage) [Two weeks and six weeks]

#### 2.1.2 Secondary outcome measures

Oral Hygiene Index [Two weeks and six weeks]
Probing depth [Two weeks and six weeks]
Gingival Index [Two weeks and six weeks]

### 2.2 Operational definition

#### 2.2.1 Gingivitis

It is defined as a site‐specific inflammatory condition initiated by dental biofilm accumulation and characterized by gingival redness and edema and the absence of periodontal attachment loss [24].

#### 2.2.2 Gingival index (Loe and Silness) [21]

1. No inflammation.
2. Mild inflammation, slight change in color, slight edema, no bleeding on probing.
3. Moderate inflammation, moderate glazing, redness, bleeding on probing.
4. Severe inflammation, marked redness and hypertrophy, ulceration, tendency to spontaneous bleeding.

### 2.3 Study design

Triple blind randomized clinical trial.

### 2.4 Study setting and duration

Dental clinic, Aga Khan University Hospital, Karachi**.** Six months after approval of synopsis by Ethical research committee of AKUH.

### 2.5 Sample size

Sample size is calculated by sample size calculator (sample size determination in health studies, WHO). Pradeep et al. [18] reported that the mean gingival index score at 6 weeks interval in Metronidazole gel group was 1.43 ± 0.27 while the mean gingival index score in Metronidazole and Chlorhexidine combination gel was 1.01 ± 0.38.

Keeping the above difference at the level of significance (α) at 5% and power of study (1-β) at 95%, we need at least 22 observations. We inflated the number to get 30 subjects per group.

Since, we have three experimental groups, so we need a total of 90 subjects.

#### 2.5.1 Sampling technique

Convenience sampling.

### 2.6 Sample selection

#### 2.6.1 Inclusion criteria

• Patients having 20 or more teeth
• Systematically healthy patient with no co-morbids
• Subjects with clinically confirmed gingivitis (bleeding on probing ≥10%)

#### 2.6.2 Exclusion criteria

• Pregnant or lactating females
• Subjects with removal or fixed dental prosthesis
• History of surgical or nonsurgical periodontal therapy in the last 6 months
• Use of antibiotic in the last 30 days
• Habit of smoking or use of smokeless tobacco
• Allergic to Metronidazole or Chlorhexidine
• Presence of any craniofacial syndrome patients
• Patients on medications that have effects on gingival conditions such as Nifedipine, Cyclosporine and Phenytoin etc.

### 2.7 Data collection procedure

All those patients who fulfilled the inclusion criteria will receive detailed information regarding the study and only those patients will be included who signed an informed consent. The study will be conducted according to the World Medical Association Declaration of Helsinki.

### 2.8 Training of the examiner

All participating investigators will be trained on the development of the trial, case selection, measurement techniques, sample collection, data compilation sheets and their precise role in the study. To evaluate the intra-examiner reliability five subjects, not involved in the study will be evaluated twice by each investigator for the measurements at the interval of one week.

### 2.9 Procedure

On the first visit after clinical examination, the specially designed baseline study proforma will be filled-in. The bleeding sites, probing depth and the gingival index score will be calculated for all the teeth (six sites per tooth).

Group A subjects will be instructed to apply a standard 0.2 % Chlorhexidine gel (clinica gel) on the marginal gingiva for the next 14 days, twice a day for 3 minutes, after morning and evening tooth brushing.

Group B subjects will be instructed to apply 0.8 % Metronidazole gel (anaerobic gel) twice daily for 2 minutes for two weeks.

Group C subjects will be instructed to apply 0.2% Chlorhexidine gel (clinica gel) on the marginal gingiva after morning tooth brushing and 0.8% Metronidazole gel (anaerobic gel) after evening tooth brushing. Subjects in these groups will receive the detailed and precise instruction and demonstrations on the diurnal alternate application of the two gels.

The application of the topical gels will be halted after 2 weeks and second clinical examination will be carried out for bleeding sites, probing depth and the gingival index score for all the teeth (six sites per tooth). Scaling & polishing of teeth in all three groups and oral hygiene instructions will be reinforced. Subjects will be recalled at 6 weeks for evaluation of gingival and oral hygiene indices. The reading will be recorded in the study proforma.

### 2.10 Randomization, blinding and treatment allocation

Subjects will be assigned to one of the three study groups using a computer generated randomization list. The recruitment of the patients will be performed by one investigator. All the measurements at the baseline and follow ups will be performed by the second investigator. Patient, the operator (until the gel will be handed over by the dental assistant), the investigator who is taking the measurement and the statistician will be blinded about the intervention groups. This will be ensured by giving codes to the intervention groups only known to the principal investigator who is not involved in the measurement and interaction with the patient.

## 3 Data analysis

Data will be analyzed using SPSS version 20. Descriptive statistics for variables such as age, baseline clinical parameters including probing depth, bleeding sites, gingival index, and oral hygiene index will be computed. An intention to treat analysis approach will be adopted. Generalized estimation equation (GEE) will be run to account for the correlated data (six probing sites per tooth) for the comparison of study arms. Friedman test will be used to assess the bleeding sites among three study groups at the baseline and endpoint. Similarly, gingival index and oral hygiene index in the three groups at baseline, after 2 weeks and 6 weeks will be compared. The level of significance will be kept at 0.05.

## 4 Trial status

Recruitment of patients is started and will be completed by the end of December 2019.

## Ethical approval and consent to participate

Ethical Approval was granted from the Ethics review committee of Aga Khan University Hospital (4577-Sur-16). The CONSORT and SPIRIT guidelines for the randomized controlled trial were followed.

## Funding

We acknowledge financial support by the department of surgery, Aga Khan University Hospital funding programme. The funders play no role in the study design, collection, management, analysis and interpretation of data or the final result and its publication, nor do they have ultimate authority over any of these actions. Funding was granted upon peer-review of this study proposal.

## Authors’ contributions

All authors have read and approved the final version of this manuscript.

## Conflicts of interest

None.

## Guarantor

Dr. Sheikh Bilal Badar.

## Research Registration Unique Identifying Number (UIN)

Clinicaltrials.gov NCT03142607.
